# Integrated Analysis of Glutathione Metabolic Pathway in Pancreatic Cancer

**DOI:** 10.3389/fcell.2022.896136

**Published:** 2022-06-03

**Authors:** Xingui Wu, Ruyuan Yu, Meisongzhu Yang, Yameng Hu, Miaoling Tang, Shuxia Zhang, Ainiwaerjiang Abudourousuli, Xincheng Li, Ziwen Li, Xinyi Liao, Yingru Xu, Man Li, Suwen Chen, Wanying Qian, Rongni Feng, Jun Li, Fenjie Li

**Affiliations:** ^1^ Program of Cancer Research, Affiliated Guangzhou Women and Children’s Hospital, Zhongshan School of Medicine, Sun Yat-Sen University, Guangzhou, China; ^2^ Department of Biochemistry, Zhongshan School of Medicine, Sun Yat-sen University, Guangzhou, China; ^3^ Department of Pediatric Surgery, Guangdong Provincial Key Laboratory of Research in Structural Birth Defect Disease, Guangzhou Women and Children’s Medical Center, Zhongshan School of Medicine, Sun Yat-Sen University, Guangzhou, China

**Keywords:** pancreatic cancer, metabolic enzyme genes, glutathione metabolism, GPX2, WGCNA

## Abstract

Metabolic enzyme-genes (MEs) play critical roles in various types of cancers. However, MEs have not been systematically and thoroughly studied in pancreatic cancer (PC). Global analysis of MEs in PC will help us to understand PC progressing and provide new insights into PC therapy. In this study, we systematically analyzed RNA sequencing data from The Cancer Genome Atlas (TCGA) (n = 180 + 4) and GSE15471 (n = 36 + 36) and discovered that metabolic pathways are disordered in PC. Co-expression network modules of MEs were constructed using weighted gene co-expression network analysis (WGCNA), which identified two key modules. Both modules revealed that the glutathione signaling pathway is disordered in PC and correlated with PC stages. Notably, glutathione peroxidase 2 (*GPX2*), an important gene involved in glutathione signaling pathway, is a hub gene of the key modules. Analysis of immune microenvironment components reveals that PC stage is associated with M2 macrophages, the marker gene of which is significantly correlated with *GPX2*. The results indicated that *GPX2* is associated with PC progression, providing new insights for future targeted therapy.

## Introduction

PC remains a highly fatal malignancy and is the fourth leading cause of cancer-related mortality in both sexes in the United States (Rahib et al., 2014; Morrison et al., 2018; Mizrahi et al., 2020), while it is the sixth leading cause of cancer-related mortality in China (Song et al., 2013; Zhu et al., 2018; Zhao et al., 2019). The incidence and mortality of PC have increased year on year. Surgical resection remains the only potentially curative treatment for PC (Sala Elarre et al., 2019). However, nearly two-thirds of patients lose the chance of surgery because of rapid progression and metastasis of PC. Even with early surgically resection, the recurrence rate is still high. For patients with locally advanced PC or distal metastasis, the 5-survival rate is also poor (Mizrahi et al., 2020). Hence, identifying new biological mechanisms and developing new strategies for PC treatment are urgently required.

Aberrant metabolism, especially redox homeostasis, is a major hallmark of cancer, and these changes promote the acquisition and maintenance of malignant properties (Gray et al., 2014). Glutathione is the most important regulator of the redox homeostasis, which can be altered by the activity of glutathione peroxidase (Roth et al., 2002; Vander Heiden et al., 2009; Levine and Puzio-Kuter, 2010; Lunt and Vander Heiden, 2011). Glutathione metabolism plays an important role in many cellular processes, including cell differentiation, proliferation and apoptosis (Ballatori et al., 2009; Cacciatore et al., 2010; Traverso et al., 2013). It was also reported that the expression of genes related to glutathione metabolism are increased in many tumors. Among them, *GPX2* is an important antioxidant enzyme in glutathione metabolism, which can scavenge a variety of peroxides (Brigelius-Flohe and Kipp, 2012; Brigelius-Flohe and Maiorino, 2013; Sakamoto et al., 2014; Huang et al., 2018). Additionally, *GPX2* is highly expressed in many tumors and can promote tumor growth (Naiki et al., 2018; Du et al., 2020; Li et al., 2020). However, little is known about the role of *GPX2* in PC.

In this study, we analyzed the RNA sequencing data from TCGA (n = 180 + 4) and GSE15471 (n = 36 + 36). We identified the differentially expressed genes (DEGs), which were then subjected to Gene Ontology (GO) (Ashburner et al., 2000) and Kyoto Encyclopedia of Genes and Genomes (KEGG) (Kanehisa and Goto, 2000) pathway enrichment analysis. It revealed that metabolic pathways are significantly activated in PC. Co-expression network modules of MEs were constructed using WGCNA (Langfelder and Horvath, 2008), which identified two key modules associated with PC. The glutathione signaling pathway is included in the two modules and correlated with PC stages. *GPX2*, identified as a hub gene of the modules, is associated with M2 macrophages and is significantly upregulated during PC progression. The findings suggest *GPX2* as a good marker of tumor heterogeneity and provides a basis for future targeted therapy.

## Materials and Methods

### The Metabolic enzymes Datasets and Patient Information Acquisition

We organized the 1689 MEs from the HMDB database (Supplementary Table S1) (Wishart et al., 2018). We used 180 cases RNA-seq of pancreatic ductal adenocarcinoma (PAAD and 4 cases of normal tissue), with clinical information for WGCNA analysis in TCGA (http://www.cancer.gov/tcga). Meanwhile, we also organized 36 normal samples and 36 tumor samples (GSE15471) of microarray from the GEO database.

### Microarray Analysis

The microarray data GSE15471 were downloaded from the Gene Expression Omnibus (GEO, http://www.ncbi.nlm.nih.gov/geo/) (Barrett et al., 2005). GSE15471 dataset contained 36 PC tissues and 36 paired adjacent normal tissues. For further analysis, the data was profiled on the GPL570 platform (Affymetrix, Human Genome U133 plus 2.0 Array). Differentially expressed genes (DEGs) were identified using the R software (version 4.0.5) package limma. The cutoff criteria were *p*_value <0.05, and |log2(fold change)(FC)| > 0.586, while RNAs with low expression values was filtered out.

### TCGA-PAAD RNA-Seq Analysis

The R software (version 4.0.5) package DEGseq (Wang et al., 2010) were used to analyze the RNA-seq from the TCGA-PAAD datasets and to identify DEGs. The cutoff criteria were *p*_value <0.05 and |log2(fold change)(FC)|>0.586, and filtered out RNAs with low expression values (the number of samples with normalized gene expression <1 was more than half of the total number of samples).

### Weighted Gene Co-Expression Network Analysis

RNA-seq and microarray datasets used above was analyzed by WGCNA to construct a co-expression network (Langfelder and Horvath, 2008). The expression profiles of MEs were obtained from the TCGA dataset and GSE15471 datasets, respectively. Then the samples were clustered through the systematic cluster tree to determine any outliers.

The soft-thresholding function was used calculate the power parameter. The dynamic tree cut method was used to identify the modules of co-expressed gene. Then a dendrogram of genes was obtained using a hierarchical clustering approach, which was based on dissimilarity of the unsigned topological overlap matrix (TOM). Lastly, genes with similar expression profiles were grouped and network modules was produced.

### Protein-Protein Interaction Network Construction

We chose two modules from the TCGA and GSE15471 by screening edges and the criterion of weight value was >0.02 for the brown module (TCGA) and the black module (GSE15471) respectively. To visualize the co-expression network and identify the nodes and hub genes, the results were input into Cytoscape to (v3.8.0; downloaded from the website https://cytoscape.org/) (Shannon et al., 2003).

### Functional Enrichment Analysis of Genes

We used the KEGG Orthology-Based Annotation System (KOBAS) (Xie et al., 2011) for GO and KEGG pathway enrichment analysis. A *p*_value <0.05 was set as the cutoff criterion.

### mRNA Expression Data Analysis Using CIBERSORT

CIBERSORT (https://cibersort.stanford.edu/index.php) (Newman et al., 2015) was used to analyze the mRNA expression data for immune microenvironment components determination.

## Results

### Metabolism Enzyme Genes Show Significantly Higher Expression Than Other Genes in Pancreatic Cancer

**FIGURE 1:**
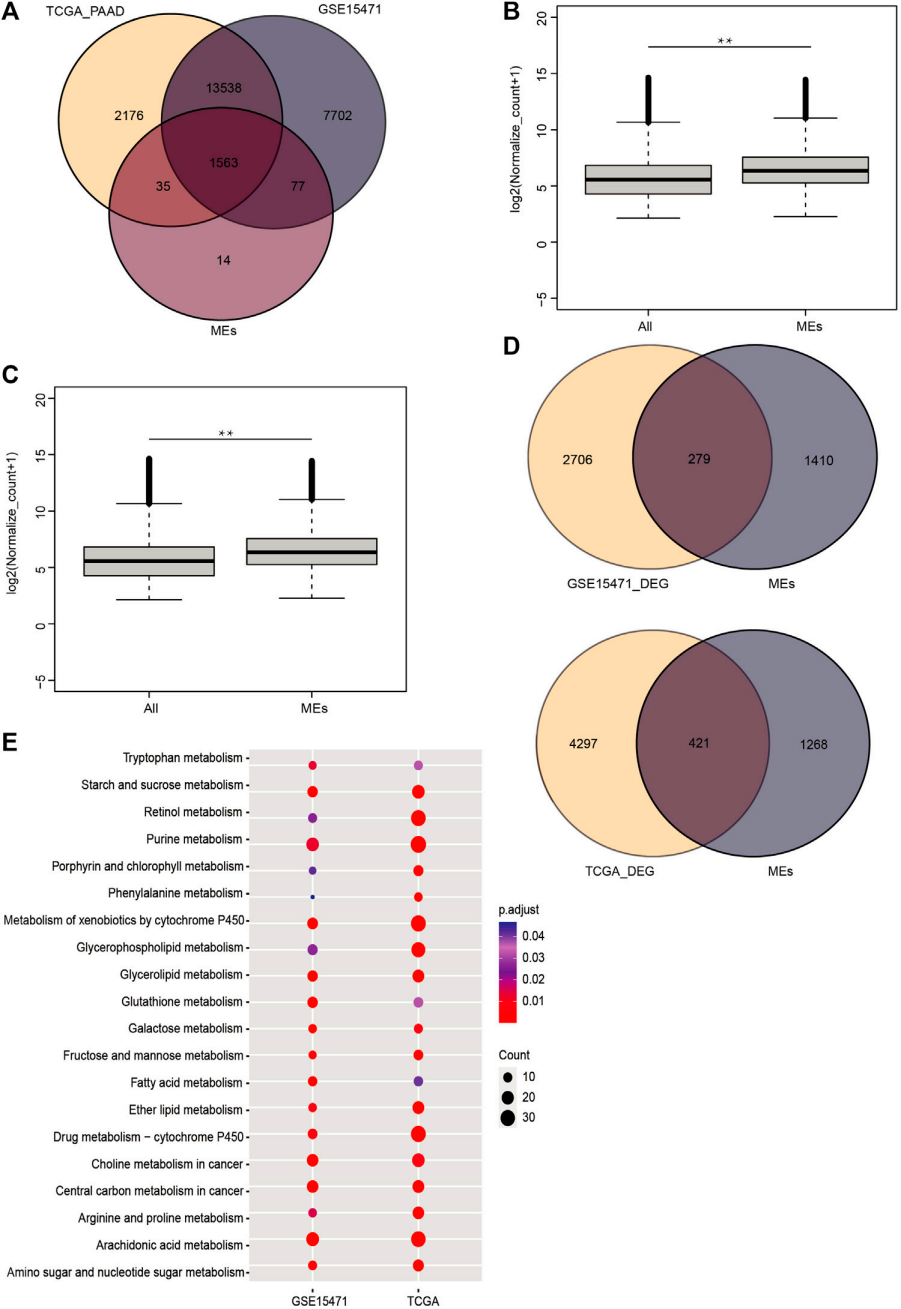
In PC, metabolic enzyme-genes are abnormally expressed. **(A)** Venn diagram of metabolic enzyme-genes in human pancreatic cancer. **(B,C)** Comparison of the expression levels of other highly expressed genes and metabolic enzyme genes in the TCGA and GSE15471 data (**, Pvalue < 0.01). **(D)** Venn diagram of differentially expressed genes and metabolic enzyme genes in the TCGA and GSE15471 data. The KEGG pathway enrichment analysis for the differentially expressed genes. **(E)** The TCGA and GSE15471 data showed that metabolic pathways, including the glutathione pathway, were enriched in both sets of data (*p* < 0.05).

We used the HMDB database and identified a total of 1689 metabolism-related genes (Supplementary Table S1). Then we extracted data for 180 samples of PAAD and 4 samples of normal tissue from the TCGA dataset and used the GSE15471 dataset comprising 36 PAAD samples and 36 normal samples. We found that 92.5% (1563) of the metabolism-related genes were expressed in both datasets (Figure 1A). The expression levels of metabolism-related genes were significantly higher than those of the other expressed genes in these datasets (*p*_value <0.01) (Figures 1B,C). Next, we identified the significantly DEGs between the PAAD and normal tissue data, which showed that 279 and 421 metabolism-related genes were significantly expressed in the TCGA and GSE15471 datasets, respectively (|log2(FC)| > 0.586 and *p*_value <0.05) (Figure 1D). KEGG enrichment analysis of both datasets showed that metabolic pathways are significantly disordered in tumor tissues of PC, including glutathione metabolism pathway (Figure 1E).

### Co-Expression Network Modules of MEs Identified by Weighted Gene Co-Expression Network Analysis in the GSE15471 Data

**FIGURE 2:**
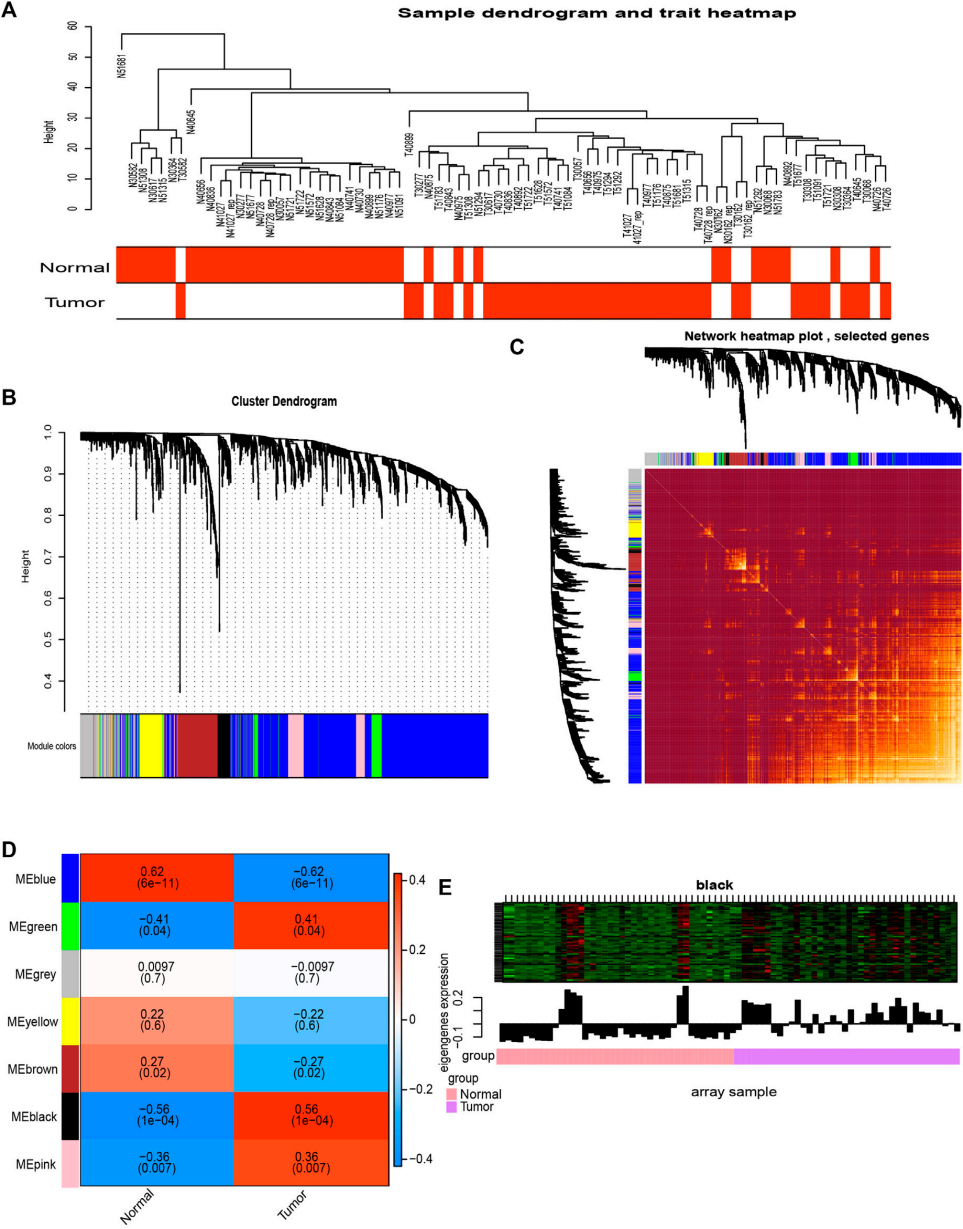
Co-expression analysis of MEs from the GSE15471 data. **(A)** Clustering tree of PC samples in the GSE15471 data. **(B)** Gene distribution in the WGCNA network analysis. **(C)** Heatmap plot of the topological overlap in the MEs network. **(D)** Analysis of the relationships between genes in modules between tumor and normal samples. **(E)** In the black module, for most genes, expression in the tumor samples was higher than that in the normal samples.

We performed WGCNA analysis on metabolic genes in GSE15471. We found that tumor tissue could be well differentiated from normal tissue using the created metabolic gene clusters (Figure 2A). We detected 7 modules in the network (Figure 2B). Genes in the seven modules showed a high correlation with each other (Figure 2C). The black module was also found to have the strongest correlation with pancreatic cancer patients, with a coefficient of correlation of 0.56 (*p* = 1 × 10^−4^) (Figure 2D). It shows that in the black module, gene expression was higher in most tumor samples than in normal tissue (Figure 2E). Therefore, this module was selected as the most clinically important screening module for further analysis.

### Co-Expression Network Modules of Metabolic Enzyme-Genes Identified by Weighted Gene Co-Expression Network Analysis in the The Cancer Genome Atlas Data

**FIGURE 3:**
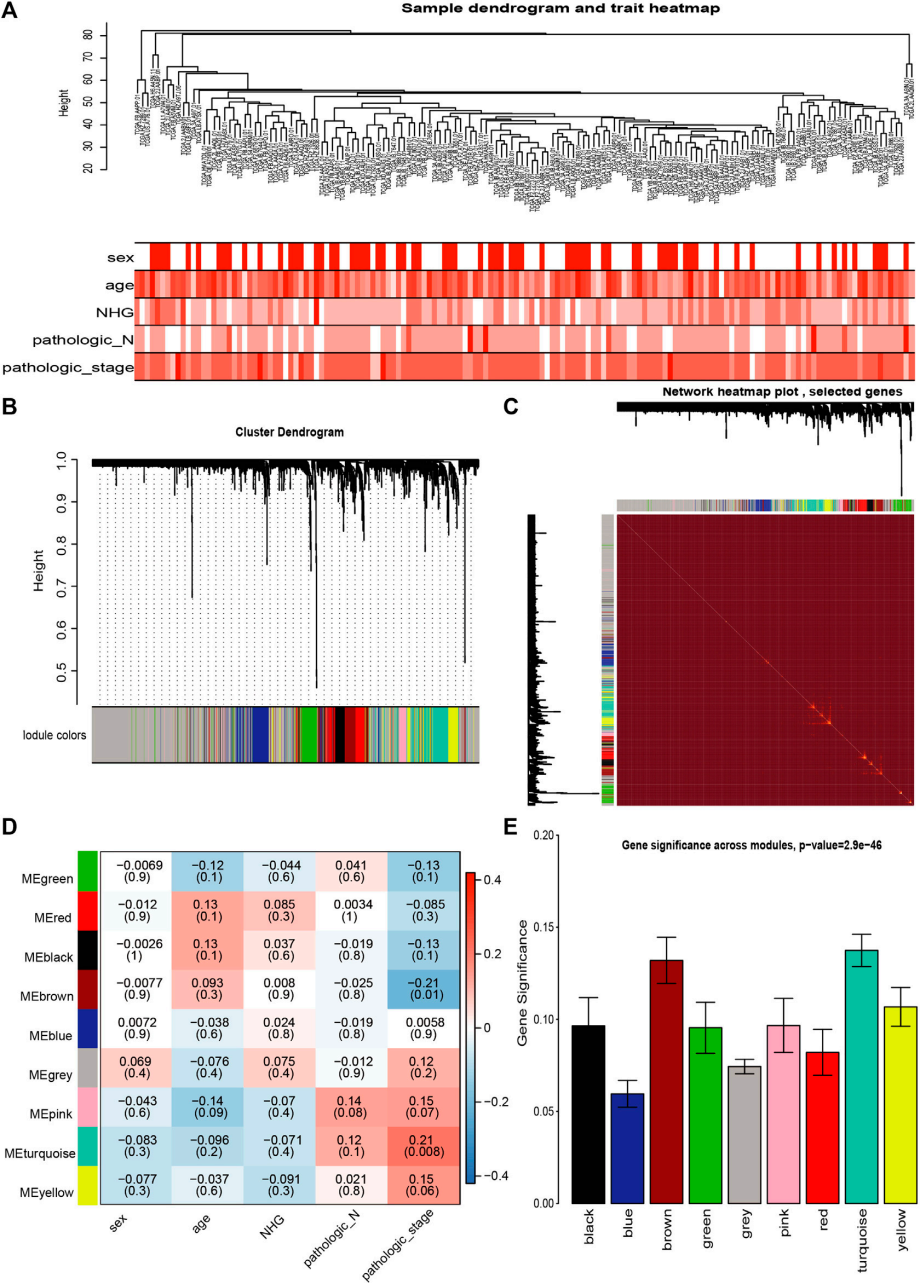
Co-expression analysis of metabolic enzymes genes from the TCGA data **(A)** Clustering tree of PC samples in the TCGA data. **(B)** Gene distribution in the WGCNA network analysis. **(C)** Heatmap plot of the topological overlap in the MEs network. **(D)** Analysis of relationships between genes in modules between tumor and normal samples. **(E)** Distribution of pathologic_stage-related genes in all modules. Genes are presented on the X-axis, and the enrichment significance is shown on the Y-axis.

We also subjected metabolic genes in the TCGA to WGCNA analysis. Since there were only four cases of normal samples of pancreatic cancer in the RNA-seq data of TCGA, we used other clinical indicators, such as sex, age, neoplasm histologic grade (NHG), pathologic N, and pathologic stage in the analysis (Figure 3A). Subsequently, we detected nine modules in the network (Figure 3B). Genes in the nine modules showed a high correlation with each other (Figure 3C). The results indicated that the brown module had the most significant correlation with pathologic_stage (correlation coefficient = −0.21, *p* = 0.008, Figure 3D). We examined the modules’ average gene significance related to pathologic stage, and the brown module was also found to have the most negetive association with the pathologic stage of PC (Figure 3E). Therefore, the brown module was selected as the most clinically important screening module for further analysis.

### Protein-Protein Interaction Network of Important Modules Reveals That the Glutathione Signaling Pathway is Dysregulated

**FIGURE 4:**
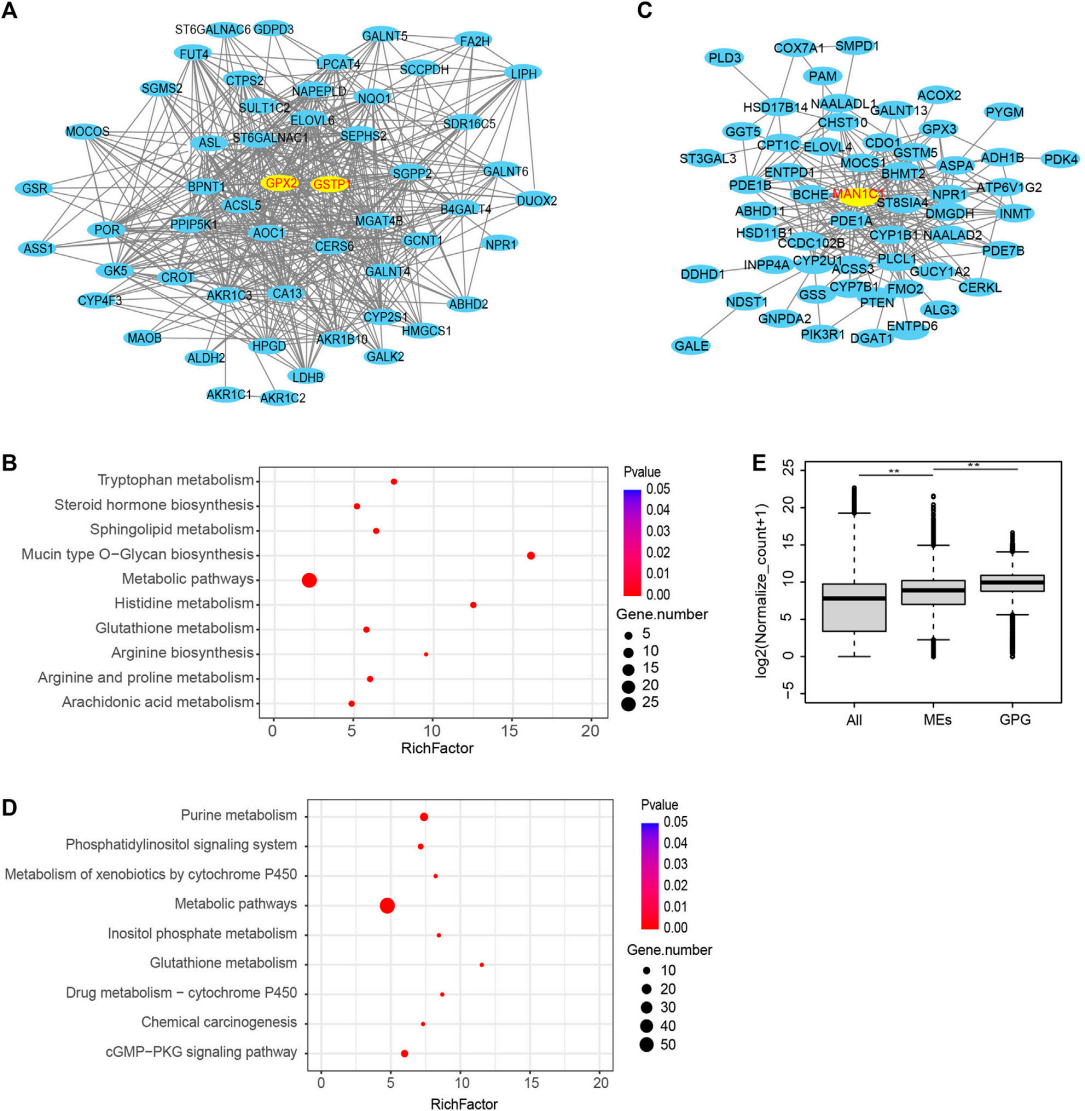
PPI network of the two modules. **(A)** PPI network was analyzed using the black module of WGCNA in the GSE15471. **(B)** The KEGG pathway enrichment analysis for the black module. **(C)** PPI network was analyzed using the brown module of WGCNA in the TCGA. **(D)** The KEGG pathway enrichment analysis for the brown module in TCGA. **(E)** Comparison of the expression levels of the GPG and other metabolic pathway genes. (**p value < 0.01).

Then the black module of the GSE15471 data was used to construct a PPI network (Figure 4A), in which *GPX2* and *GSTP1* (encoding glutathione S-Transferase Pi 1) were identified as the hub genes. The black module was analyzed by KEGG, which showed that the glutathione pathway was significantly enriched (Figure 4B). Then we also used the brown module of TCGA to create a PPI network in which the hub genes was *MAN1C1* (mannosidase alpha class 1C member 1) gene (Figure 4C). The brown module was then analyzed by KEGG, which showed that the glutathione pathway was also significantly enriched (Figure 4D). We then compared the expression levels of the metabolic enzyme genes and found that the gene expression levels of members of the glutathione pathway were significantly higher than those of other metabolic genes (Figure 4E). These results indicated that the glutathione pathway are significantly dysregulated in pancreatic cancer.

### Glutathione Signaling Pathway Genes are Associated With PC Stages

**FIGURE 5:**
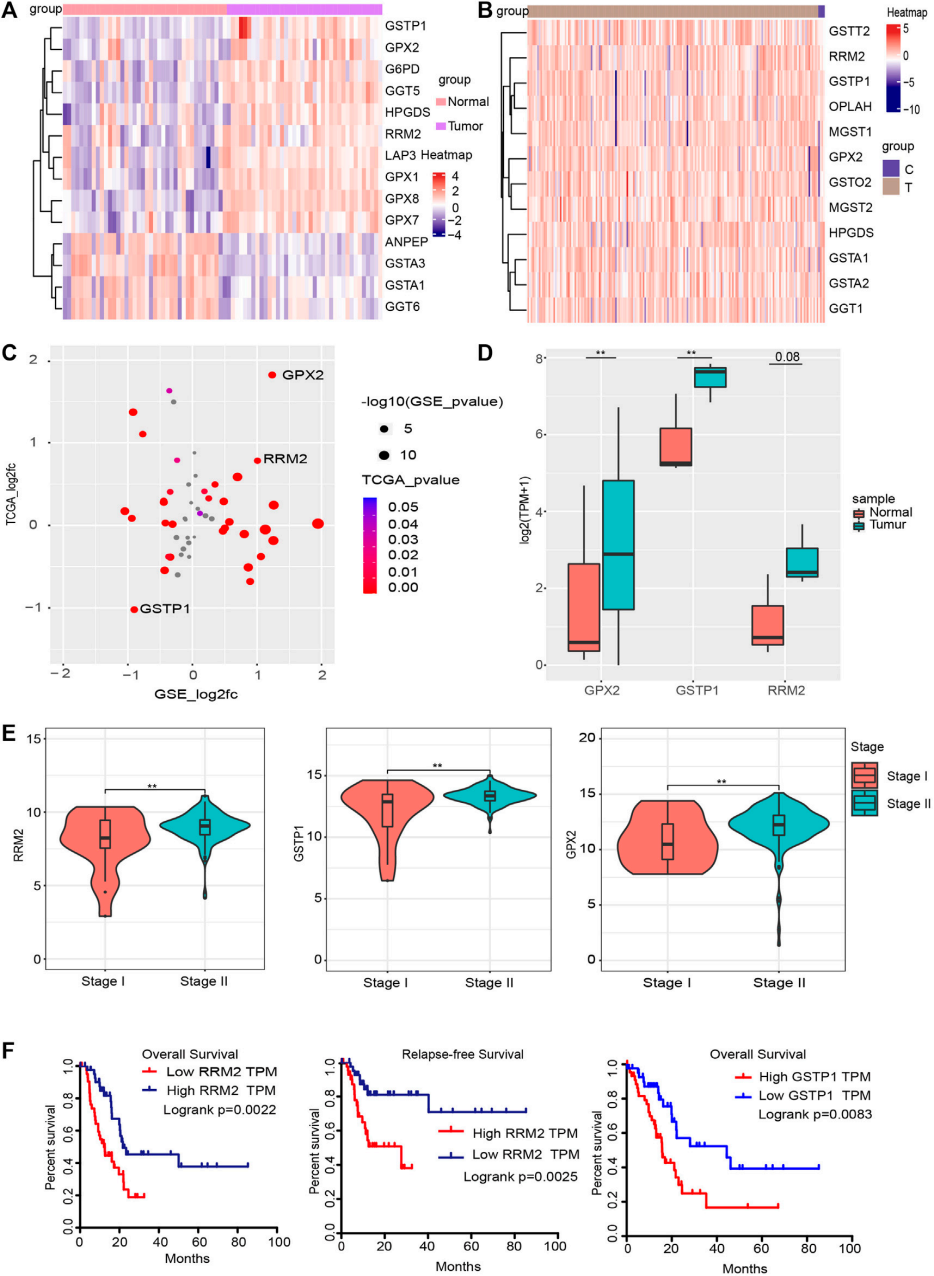
Glutathione signaling pathway genes are associated with PC stage. **(A,B)** Heatmap of differentially expressed glutathione pathway genes in the GSE15471 and TCGA datasets. **(C)** Four quadrant diagram of the glutathione genes that differ between the GSE15471 and TCGA datasets. **(D)** The expression level of RRM2, GSTP1 and GPX2 in our own RNA-seq data (**p value < 0.01). **(E)** In the TCGA data, the RRM2, GSTP1 and GPX2 genes were significantly differently expressed in different stages of PC (*p* < 0.05). **(F)** Survival analysis and Relapse-free Survival analysisof RRM2 (left and middle) in TCGA dataset; survival analysis of GSTP1 in TCGA dataset (right).

Since glutathione pathway has a great impact on the clinical evaluation of PC, we examined the expression level of genes involved in glutathione pathway. In the GSE15471 data, 14 genes in the glutathione pathway were differentially expressed (|log2(FC)| >0.586, *p*_value <0.05) (Figure 5A). In the TCGA, 12 glutathione pathway genes were found to be significantly differentially expressed (Figure 5B). We found that glutathione pathway genes, including GPX2, GSPT1, RRM2, are differentially expressed in both data (Figure 5C). We also verified the expression of GPX2, GSTP1 and RRM2 in glutathione pathway in our own RNA sequencing data and the results was consistent with that of public data we used (Figure 5D). Among them, *GPX2*, *GSTP1* and *RRM2* (Ribonucleotide Reductase Regulatory Subunit M2) showed different expression levels in different stages of PAAD (Figure 5E) (**p_value < 0.01). In stage II, the expression levels of these three genes were relatively high, suggesting they could be clinical indicators and providing a preliminary basis for later tumor heterogeneity. Then we performed the survival analysis of the glutathione pathway genes in patients with PC. The results indicated that patients with higher expression of glutathione pathway genes, including GSTP1 and RRM2, have shorter survial term (Figure 5F). Collectively, these data indicated that glutathione pathway genes may play critical roles in PC.

### *GPX2* is Associated With M2 Macrophages, Which Predicts Cancer Immune Heterogeneity

**FIGURE 6:**
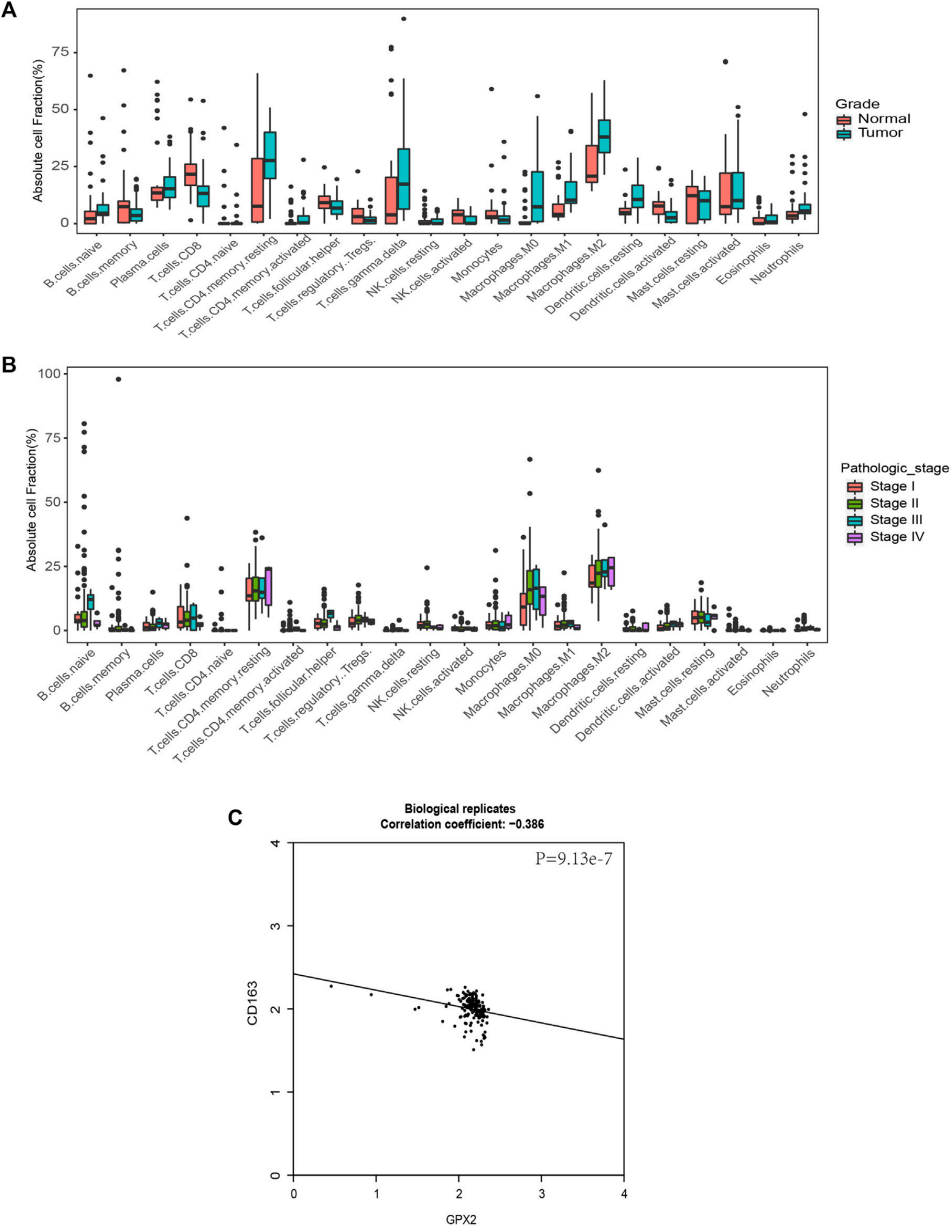
*GPX2* is associated with M2 macrophages, which predicts cancer immune heterogeneity. **(A)** Comparison of immune cell fractions among subtypes. Immune cell fraction distribution in 36 normal samples and 36 pancreatic cancer groups. **(B)** Comparison of immune cell fractions between subtypes. Among them, 21 samples in Stage I, 151 samples in Stage II, 4 samples in Stage III and 5 samples in Stage IV, with a simple row score >0.05. **(C)** Scatter plots showing the correlation between *GPX2* and *CD163*.

Since glutathione pathway genes, including *GPX2*, showed different expression levels in different stages of PAAD, we tried to predict immune heterogeneity based on gene expression profiles in PC at different stages. To comprehensively determine the cellular components of the tumor microenvironment across different stages of pancreatic cancer, we used the CIBERSORT in silico cytometry method (Newman et al., 2015) to evaluate 22 different immune cell types in 36 normal tissues and 36 tumor tissues in GSE15471, and 153 tumor tissues at different stages in TCGA. In the results for the GSE15471 data, PC appeared to promote an M2 macrophage gene signature compared with normal pancreatic tissue (Figure 6A). In the results of the TCGA analysis, M2 macrophages were more abundant in patients with stage IV disease compared with the patients at other stages (Figure 6B). We evaluated the correlation between the expression of *GPX2* and *CD163*, the marker of M2, and it showed they are significantly correlated (Figure 6C). These results suggested that *GPX2* may play important roles in cancer immune heterogeneity.

## Discussion

In the present study, we analyzed TCGA and GSE15471 data and found significant differences in glutathione pathway gene expression in PC. In normal tissues, the expression levels of GPX2, GSTP1 and RRM2 were lower than those in PC cells. Then, using the TCGA data, we found that the expression levels of GPX2, GSTP1 and RRM2 were also different in different stages of PC. In addition, these three genes showed higher expression in stage II than stage I samples. These results are also consistent with the findings of previous studies.

Transcriptome profiling and microarray of tumor samples are widely used to interrogate pathway functionality and for phenotype-based patient classification. In our study, we found that there are many M2 macrophages around PC cells. To develop markers for PC staging, further single-cell sequencing is required to finely dissect the expression of glutathione pathway genes in PC cells.

In conclusion, bioinformatic analyses revealed that eight genes of the glutathione pathway are differentially expressed between PC and normal pancreas tissues, and three of them show significant expression differences in different stages of PC staging. These results help to refine the cellular heterogeneity of PC. Exploiting such cellular heterogeneity, targeted drugs are used to kill early PC cells at a later stage. This can prevents pancreatic cancer from progressing, thereby prolonging the survival of patients with PC and buying time for the development of further treatment.

## Data Availability

The original contributions presented in the study are included in the article/Supplementary Material, further inquiries can be directed to the corresponding authors.

## Abbreviations

MEs, Metabolic enzyme-genes; *GPX2*, Glutathione Peroxidase 2; *GSTP1*, glutathione S-Transferase Pi 1; *RRM2*, Ribonucleotide Reductase Regulatory Subunit M2; PC, Pancreatic cancer; WGCNA, weighted gene co-expression network analysis; TCGA, The Cancer Genome Atlas; GO, Gene Ontology; KEGG, Kyoto Encyclopedia of Genes an Genomes; HMDB, Human Metabolome Database; DEGs, differentially expressed genes; NHG, degree of cancer cell abnormalities; *CD163*, CD163 molecule; FC, fold change.
